# Fatal cardiac arrest in an adult patient with euthyroid anti-SSA/Ro-positive connective tissue disease: a case report

**DOI:** 10.1186/1755-7682-2-15

**Published:** 2009-05-15

**Authors:** Michele Adami, Michele Nardin, Enrico Morello, Claudio Crivellaro, Christian J Wiedermann

**Affiliations:** 1 Department of Internal Medicine, Central Hospital of Bolzano, Lorenz-Böhler-Street 5, IT-39100 Bolzano, Italy; 2 Division of Anaesthesiology and Intensive Care Medicine 2, Central Hospital of Bolzano, Lorenz-Böhler-Street 5, IT-39100 Bolzano, Italy; 3 Department of Haematology, Central Hospital of Bolzano, Lorenz-Böhler-Street 5, IT-39100 Bolzano, Italy

## Abstract

**Background:**

Until recently, anti-SSA/Ro antibodies were not considered pathogenic for severe heart disease in adults. Prolongation of the mean QTc interval in electrocardiograms of adult patients with anti-SSA/Ro-positive connective tissue disease has been reported and could contribute to complex arrhythmias in such patients. Furthermore, complete heart block may also be related to these autoantibodies.

**Case presentation:**

We describe the occurrence of fatal complete heart block in a euthyroid adult patient with undifferentiated connective tissue disease and polyglandular autoimmune syndrome type 2 associated with cardiovascular autonomic dysfunction who had normal QTc interval. The patient's serum contained anti-SSA/Ro.

**Conclusion:**

This case might indicate that, although the adult heart conduction system may be relatively resistant to the development of anti-Ro-associated complete heart block, cardiac arrest may develop and even be fatal.

## Background

The role of anti-SSA/Ro antibodies in the development of congenital heart block is now established [1]. Until recently, anti-SSA/Ro antibodies were not considered pathogenic for severe heart disease in adults. In mixed (M) connective tissue disease (CTD), minor cardiac rhythm abnormalities appear in approximately half of the adult patients [2]. Prolongation of the mean QTc interval in electrocardiograms of adult patients with anti-SSA/Ro-positive CTD has been reported and could contribute to complex arrhythmias in such patients [3]. Furthermore, complete heart block may also be related to these autoantibodies [4-6]. We report a patient with cardiovascular autonomic dysfunction and newly diagnosed undifferentiated (U) CTD in autoimmune polyglandular syndrome type 2 (APS-2) who developed complete atrioventricular block with fatal outcome.

## Case presentation

A 37-year-old woman with known autoimmune thyroiditis and primary adrenal insufficiency of APS-2 was admitted 6 months after being diagnosed with newly developed Raynaud's phenomenon affecting the hand. Malaise, arthralgias, muscle weakness, low-grade fever and generalized lymphadenopathy had developed and persisted despite sufficient hormone replacement with thyroxin and cortisone acetate. She tolerated exposure to ultraviolet light. Medication history included no drugs typically known to trigger CTD nor was she exposed to silica or cigarette smoke. There were no seizures or other neuro-psychiatric symptoms in the past.

Examination disclosed total hand edema in addition to generalized brown hyperpigmentation. Vital signs were normal and there were no signs and symptoms of severe renal, cardiac, pulmonary or central nervous system disease. Cough, dyspnea or pleuritic chest pain was absent. Chest x-ray and electrocardiogram were unremarkable. The QTc interval was normal at the electrocardiogram. Results of laboratory tests are given in Table 1. The patient displayed positivity for anti-SSA/Ro antibodies. Serum electrophoresis showed polyclonal hypergammaglobulinemia, negative for monoclonality on immune fixation; in bone marrow biopsy, hyperplasia was found predominantly of normal granulopoiesis and mild infiltration of B lymphocytes of about 8%; anti-Sm antibodies were only transiently positive on admission (negative after two additional weeks); in morphological and immunophenotypic analyses of supraclavicular and abdominal lymph node biopsies, follicular hyperplasia was seen which was interpreted as reactive lymph node changes. Normal fT4 excludes clinical hypothyroidism. Reduced fT3 and slightly elevated TSH levels indicate presence of the euthyroid sick syndrome.

**Table 1:** Laboratory studies

Parameter	Level	Normal
TSH	13.83 mIU/L	0.30 – 3.94
T3	1.89 pg/mL	2.42 – 4.36
ACTH	1601 pg/mL	5.0 – 47.0
Hematocrit	29.7%	37 – 52
Hemoglobin	9.9 g/dL	12 – 18)
Immunoglobulin G	4040 mg/dL	751 – 1560
Kappa light chains	48.7 mg/dL	absent
Direct Coombs'	positive	negative
Beta-2 microglobulin	4.2 mg/L	1.0 – 1.9
D-dimer	2527 ng/ml	<500
Factor V Leiden mutation	heterozygote	absent
ANA	> 1/1280 "speckled"	absent
RNP/Sm	positive	negative
SS-A/Ro	positive	negative

*Test results within normal range*: electrolytes (including calcium), glucose, kidney and liver function tests, white blood cell count with differential and platelets, free T4, IgM, IgA, indirect Coombs' test, erythrocyte sedimentation rate, C-reactive protein, troponin T; global coagulation parameters; urine analysis, urinary free lambda light chains; anti-extractable nuclear antigens antibodies (SS-B/La, Scl-70 and Jo-1), anti-dsDNA; anti-mitochondrial (AMA), anti-neutrophil cytoplasmic (ANCA) against myeloperoxidase (MPO) and proteinase-3 (PR3), anti-liver kidney microsomal, anti-parietal cell, anti-transglutaminase IgA and IgG, and anti-cardiolipin IgG and IgM antibodies; rheumatoid factor; angiotensin converting enzyme; complement levels C3 and C4; candida culture, serology on single determination for hepatitis viruses A, B and C, human immunodeficiency virus antigen/antibody, cytomegalovirus, herpes simplex virus-1 and -2, and varicella-zoster virus. Elevated ACTH reduced within days to normal values upon administration of increased doses of cortisone acetate resulting in cortisol levels of around 400 ng/mL (normal range: 65 – 250).

*Abbreviations*: ANA, antinuclear antibodies; RNP/Sm, combined nuclear ribonucleoprotein Smith antigen.

Ambulatory electrocardiography for 24 hours showed a heart rate variability that was pathologically reduced. Orthostatic hypotension was symptomatic in the absence of volume depletion. Echocardiography confirmed near-normal left ventricular function and excluded structural heart disease. The patient received hormone replacement with thyroxin and intravenous hydrocortisone (240 μg per day); omeprazol 40 mg and thrombosis prophylaxis with enoxaparin 40 mg were given daily.

Before initiation of therapy for CTD, the patient became unresponsive on the general ward with no normal breathing and no signs of circulation. Sudden cardiac arrest occurred unwitnessed with cessation of cardiac activity of unknown duration. On emergency ECG monitoring, wide QRS escape rhythm was seen of frequency < 20 bpm with increasing periods of asystole. Complete heart block was successfully reversed to spontaneous circulation by cardiopulmonary resuscitation with chest compression, and administration of atropine and adrenaline. Severe anoxic brain injury was subsequently diagnosed (corneal and pupillary light reflexes absent for 24 hours; Glasgow Coma Scale score of 3 after 48 hours). When complete heart block developed, electrolytes were normal, serial troponin T measurement ruled out acute coronary syndrome and post-resuscitation 12-lead ECG did not show sign of ischemia or myocarditis. The patient progressed to a persistent vegetative state and, just 2 months later, died a natural death in a long-term care facility of a repeat episode of cardiac arrest.

## Discussion

UCTD was diagnosed as defined by Raynaud's phenomenon with swollen hands and highly positive anti-RNP antibody titer, associated with autoimmune conditions including hypergammaglobulinemia, reactive lymphadenopathy, Coombs'-positive anemia, and APS-2. The patient had cardiovascular autonomic dysfunction as evidenced by symptomatic orthostatic hypotension in the absence of volume depletion as well as significantly reduced heart variability on ambulatory electrocardiography. Following development of complete heart block, electrophysiological studies and eventual pacemaker insertion were not performed because of severe neurological disability and poor prognosis.

Cardiac arrhythmias have been described in autoimmune diseases with antibodies causing inflammatory myopathies [7]. In the current patient, anti-SSA/Ro antibodies may well have contributed to the development of sudden cardiac arrest. A high titer of anti-RNP antibodies in patients with UCTD is a powerful predictor of later evolution into mixed (M)CTD [8], for which anti-Ro/SSA-mediated non-fatal complete heart block has been reported as rare event [1,4-6]. The patient reported here did not yet fulfil diagnostic criteria for MCTD. As she was positive for both anti-RNP and anti-SSA, however, the presence of anti-RNP antibodies could be predictive of later development of MCTD.

Signs of an autonomic nervous system dysfunction involving the cardiovascular system can be discerned in the majority of anti-SSA/Ro- and anti-SSB/La-positive patients with CTD [3]; on this basis, a putative role for cardiovascular autonomic dysfunction in favoring an adverse outcome of complete heart block by impairment of compensatory shock mechanisms as was seen in the present patient, cannot be ruled out. Moreover, it should be underlined that cardiovascular autonomic dysfunction was similarly detected in anti-SSA/Ro-negative CTD patients [3], as it is present in other autoimmune diseases as well [9], thus suggesting that it is a feature characteristic of the disease independently of the presence of such autoantibodies.

Hypothyroidism at the moment of the cardiac arrest may be a feature of peculiar pathogenetic relevance. In fact, in patients with hypothyroidism (also subclinical), severe atrioventricular block of different degrees disappeared after supplementation with thyroxine [10,11]. Moreover, Spence et al. [12] demonstrated that maternal hypothyroidism increased markedly the risk (about 9-fold) of delivering a child with complete congenital heart block in women with circulating anti-SSA/Ro antibodies. Finally, Baumgart et al. [13] described the case of a complete heart block developing in a woman affected by anti-60 kD SSA/Ro-positive Sjögren's syndrome with concomitant hypothyroidism and hypoadrenalism secondary to hypopituitarism. The patient, early implanted with a VVI pacemaker, was then treated with a substitution regimen with prednisolone and thyroxine resulting in the restoration of the sinus rhythm. On the basis of these data, in accordance with a very recent review on this topic [1], it is possible to hypothesize that the conduction system of adult heart may display, rather than an absolute resistance, a different degree of vulnerability to anti-SSA/Ro antibodies with respect to fetal heart.

Two fundamental pathophysiological mechanisms have been proposed to account for autoantibody-mediated arrhythmogenic effects: (a) a direct mechanism, in which cardiomyocytes, and their electrophysiological properties, constitute the primary target of the autoantibodies; (b) an indirect mechanism, in which the autoantibody-mediated immune response involves the cardiac electrophysiology only secondarily [14]. The latter is the case of anti-cardiomyocyte autoantibodies producing a myocardial damage leading to the development of dilated cardiomyopathy and heart failure, which eventually represent the major cause of severe ventricular arrhythmias), or autoantibodies targeting structures indispensable for the myocardial homeostasis such as nerves and vasculature (references in [14]). Given the absence of both clinical and objective findings suggesting dilated cardiomyopathy in the case presented here, a direct mechanism most likely accounted for bradycardia.

In the context of predisposition, additional concomitant factors (such as hypothyroidism) may be able to precipitate the onset of severe heart block. The patient of the present case report was not in a hypothyroid state at the moment of heart block development as fT4 levels were normal because sufficiently substituted with thyroxine. TSH was slightly elevated and fT3 was low which is compatible with euthyroid sick syndrome [15].

Differential diagnoses of bradycardia and complete heart block have to be reasonably ruled out. The patient presented here did not assume medications potentially affecting the conduction tissue; clinical, laboratory and ECG data suggesting structural heart disease were normal, making underlying myocarditis and acute myocardial infarction unlikely, putatively resulting from coronary vasculitis or accelerated atherosclerosis, nor were echocardiographic signs of cardiac amyloidosis present in consideration of the concomitant cardiovascular autonomic dysfunction evident.

The fact that a post-mortem examination has not been performed in the patient is a clear limitation of the study.

## Conclusion

CTD may accompany APS-2 and in such patients should be considered in the differential diagnosis of otherwise unexplained symptomatology. The presence of anti-Ro/SSA-antibodies may cause atrio-ventricular conduction disturbance and bradycardia, the clinical significance of which can be evaluated by ambulatory electrocardiography for 24 or 48 hours. The conduction system of adult heart may display a different degree of vulnerability to anti-SSA/Ro antibodies and concomitant risk factors including hypothyroidism.

## Consent

An informed consent was not obtained for publication of this case report after the patient died.

## Competing interests

The authors declare that they have no competing interests.

## Authors' contributions

MA and CW involved in writing the paper with data collection and literature search. MA and MN, registrars were involved in treating the patient and data collection. EM and CC, consultants, were involved in treating the patient.
